# Factors Affecting Sleep and Wakefulness in People with Epilepsy: A Narrative Review

**DOI:** 10.3390/medicina61061000

**Published:** 2025-05-28

**Authors:** Dovydas Burkojus, Giedrė Gelžinienė, Evelina Pajėdienė, Gineta Stankevičienė, Valdonė Misevičienė, Giedrė Jurkevičienė

**Affiliations:** 1 Department of Neurology, Medical Academy, Lithuanian University of Health Sciences, LT-50161 Kaunas, Lithuania; giedre.gelziniene@lsmu.lt (G.G.); evelina.pajediene@lsmu.lt (E.P.); gineta.stankeviciene@lsmu.lt (G.S.); giedre.jurkeviciene@lsmu.lt (G.J.); 2 Hospital of Lithuanian University of Health Sciences Kauno klinikos, Department of Neurology, Epilepsy Center Affiliated Member of EpiCARE, LT-50161 Kaunas, Lithuania; 3 Department of Pediatrics, Medical Academy, Lithuanian University of Health Sciences, LT-50161 Kaunas, Lithuania; valdone.miseviciene@lsmu.lt; 4 Department of Pediatrics, Hospital of Lithuanian University of Health Sciences Kauno Klinikos, LT-50161 Kaunas, Lithuania

**Keywords:** epilepsy, sleep, circadian, sleep–wake rhythm, sleep structure, clock genes, seizures, antiseizure medication

## Abstract

The importance of sleep has been reported for decades. Epilepsy is a heterogeneous disorder comprising multiple elements that might influence sleep and wakefulness. Notably, animal studies show disruptions of the circadian molecular system in different models of epilepsy, along with altered rest–activity and other circadian rhythms. So far, studies of molecular circadian systems in people with epilepsy are lacking, prompting further research. Seizures—the primary and most debilitating symptom of epilepsy—and interictal activity disrupt regular sleep and sleep–wake rhythms. Alterations in one’s sleep structure are seen in both drug-naïve and drug-resistant patients with epilepsy. In particular, low sleep efficiency, a reduction in total sleep time, and changes in sleep stages were found in both homogenous and mixed samples of epilepsy patients. Both ictal and interictal activity were also shown to be associated with changes in peripheral circadian phase biomarkers such as melatonin and cortisol. Moreover, epilepsy comorbidities, antiseizure medications, and a variety of syndromes can be a cause of sleep problems or even sleep disorders. Sleep disorders vary depending on various comorbidities and syndromes, and encompass all major groups of sleep disorders defined in the International Classification of Sleep Disorders. Controversial findings on the effects of various antiseizure medications were found in the literature. However, medications such as benzodiazepines, gabapentinoids, and barbiturates are particularly associated with excessive daytime sleepiness. Overall, a sleep evaluation must be included in the management of every patient with epilepsy.

## 1. Introduction

Sleep is considered one of the pillars of health. Irregular sleep, more so than sleep duration, is a strong predictor of premature mortality from various causes, including cancer and cardiovascular disorders [1]. The disruption of sleep–wake rhythms can be caused by internal factors that affect circadian rhythms, such as neurodegenerative disorders and visual problems, as well as external factors like behavioral issues or certain medications [2]. People with epilepsy tend to report a worse sleep quality and excessive daytime sleepiness [3], prompting additional research into the matter. Numerous factors contribute to the overall health issues in individuals with epilepsy. These factors can affect both sleep and wakefulness and can either be unmodifiable, such as genetic predispositions, or modifiable, such as antiseizure medication.

**Figure 1:**
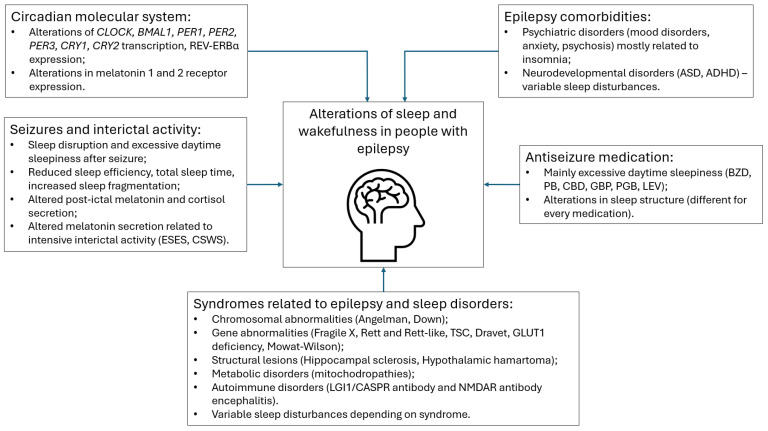
Overview of disturbances related to sleep and wakefulness in people with epilepsy. Abbreviations: CLOCK—circadian locomotor output cycles kaput; BMAL1—Basic helix-loop-helix ARNT-like protein 1; PER—period; CRY—Cryptochrome Circadian Regulator 1; ESES—electric status epilepticus in sleep; CSWS—continuous spikes and waves during sleep; TSC—tuberous sclerosis complex; ASD—autism spectrum disorder; ADHD—attention deficit hyperactivity disorder; BZD—benzodiazepines; PB—phenobarbital; CBD—cannabidiol; GBP—gabapentin; PGB—pregabalin; LEV—levetiracetam.

Given that epilepsy is a heterogeneous disorder, it is safe to assume that sleep–wake profiles are different in each individual with epilepsy. In this narrative review, we explore the factors that might influence sleep and wakefulness in people with epilepsy. In particular, we present an overview of the current evidence regarding molecular circadian system alterations in the context of seizures, the influence of seizures and interictal activity on sleep and circadian rhythms, and the influence of various syndromes, comorbidities, and antiseizure medications on sleep and wakefulness (Figure 1).

## 2. Materials and Methods

A comprehensive search was carried out across the MEDLINE (PubMed), Google Scholar, and ScienceDirect databases. The searches were performed using specific combinations of keywords: “epilepsy”, “seizures”, “sleep”, “circadian”, “sleep-wake rhythm”, “sleep structure”, “sleep architecture”, “antiseizure medication”, “anxiety”, “mood disorders” and, “psychosis”. The rest of the keywords included syndromes and etiologies associated with epilepsy and different groups or individual antiseizure medications. Further, titles and abstracts were screened for relevance. Full-text studies were assessed based on the criteria provided below.

Studies were included if they focused on disturbances or alterations in sleep, wakefulness, sleep–wake rhythms, circadian rhythms, sleep structure in people or animals with epilepsy, and epilepsy-related factors—comorbidities, syndromes, and antiseizure medications. Articles were included if they were published in English and were original research articles, systematic reviews, meta-analyses and observational studies. We included animal studies when human studies were scarce. Most of the animal studies are presented in Section 3. The search considered studies published from 1984 until April 2025. We included some historical studies from the 1990s and 1980s due to their relevance to the topic and lack of similar studies in the present day.

Studies were excluded if they were written in a language other than English or were duplicates from other databases. Editorials, letters, commentaries, and other articles that were not peer-reviewed were also excluded. Studies not examining thematic areas described previously were omitted.

The selected studies were analyzed, and data regarding disturbances or alterations in sleep, wakefulness, sleep–wake rhythms, circadian rhythms, and sleep structure in people or animals with epilepsy and epilepsy-related factors (comorbidities, syndromes, and antiseizure medications) were extracted. Key studies were organized in summary tables.

## 3. Molecular Circadian System and Epilepsy

The master circadian pacemaker is in the suprachiasmatic nucleus (SCN) of the ventral hypothalamus and transmits the photic input of the light–dark cycle to peripheral clocks in the rest of the brain and the body [4]. These rather autonomous clocks can be entrained via environmental factors to maintain the body’s rhythmicity in various processes, including the sleep–wake cycle [5]. The internal molecular circadian rhythm is maintained by regulators that positively or negatively affect the transcriptional or translational processes. Proteins such as BMAL1 and CLOCK, encoded in genes *BMAL1* and *CLOCK*, respectively, are the core transcription factors that have a variable influence on the mammalian circadian gene network, which in turn is linked to the regulation of cell metabolism and physiology at different times in the circadian rhythm [6]. Important pathways of the metabolism associated with epilepsy, such as pyridoxal, mTOR, and redox state, are linked to the molecular circadian system [7]. An overview of studies exploring the alterations in the circadian molecular system in animal models of epilepsy is presented in Table 1.

Given the heterogeneity of epilepsy, it might be expected that different epilepsy models, although to a varying degree, exhibit altered clock gene expressions and changes in circadian rhythms. A mesial temporal lobe epilepsy model showed circadian phase alterations and fragmentation in post-*status epilepticus* (SE), along with altered temporal transcriptions of *Bmal1*, *Cry1*, *Cry2*, *Per1*, *Per2*, and *Per3* in baseline, early post-SE and epileptic conditions [8], indicating the possible involvement of clock genes during epileptogenesis. Additionally, in a pilocarpine-induced epilepsy mouse model, it was observed that core body temperature oscillations were significantly lower in epileptic mice, indicating circadian disruption [9]. The same study showed a total decrease in the relative expressions of *Bmal1*, *Clock*, *Cry1*, and *Cry2* and an increase in *Per1* and *Per2* mRNA in the SCN and hippocampus [9]. Moreover, lesions in the SCN were associated with an increase in seizures via diminished GABAergic signaling [9]. This study shows a possible disruption of the circadian system in epilepsy and associations between the impairment of the circadian system and the worsening of seizures. Indeed, in a model of *Bmal1* knockout mice, the diurnal seizure threshold was found to be significantly diminished [10]. REV-ERBα is another regulator in the mammalian circadian system and controls *BMAL1* transcription [11]. Rev-erbα ablation was associated with a reduction in seizures via diminished GABA reuptake. Interestingly, the disruption of the circadian rhythm in a jet-lag-type manner was associated with a greater resistance to seizures [12]. A study of the *Kcna1*-null epilepsy mouse model revealed diminished oscillations of *Clock*, *Per1*, and *Per2* in the hypothalamus and arrhythmic rest–activity patterns [13]. Importantly, no associations were found between changes in sleep parameters and the burden of seizures, indicating that in this epilepsy model, the disruption of the molecular circadian system rather than seizures affected sleep–wake rhythms [13]. Taken together, these studies suggest that the circadian system could be significantly disrupted in epilepsy, and that this is associated with an increase in seizure burden and the alteration of rest–activity rhythms.

A study conducted by Yamakawa and colleagues revealed the dysregulation of the core circadian clock genes *Per1*, *Cry1*, *Clock*, and *Bmal1* in the hippocampus, hypothalamus and peripheral tissues such as the liver and small intestine in a mouse model of absence epilepsy [14]. Meanwhile, in a model of drug-resistant temporal lobe epilepsy, the dysregulation of *Cry1*, *Clock*, and *Bmal1* was more evident in the hypothalamus and liver, along with *Cry1* and *Clock* diurnal dysregulation in the hippocampus [14]. This not only shows the distinct involvement of the circadian molecular system in different types of epilepsy, but also the possible desynchronization of central and peripheral clocks.

Melatonin, also called “a dark hormone”, acts as a synchronizer of the body’s internal clock with light–dark cycles and has receptors in various parts of the brain [15]. A mouse model of temporal lobe SE showed increased mRNA expressions of melatonin 1 and melatonin 2 receptors in the hippocampus at 2 and 11 h post SE and 5 and 11 h post SE, respectively. Both receptors’ mRNA levels were reduced later compared to the levels a few hours post SE [16]. Although the effects of changes in the post-seizure melatonin receptor on the sleep–wake rhythm are yet to be seen, the increase in melatonin receptors in post-SE might be related to its potential seizure-reducing effects and neuroprotective properties [17].

Data on alterations in the circadian molecular system in human tissues are scarce. However, the *CLOCK* gene was found to be under-expressed in the brain tissue of tuberous sclerosis and focal cortical dysplasia patients with treatment-resistant epilepsy, and plays an important role in the excitatory and inhibitory neuron excitability threshold [18,19]. Meanwhile, CLOCK and REV-ERBα were found to be increased in the brain tissue of patients with temporal lobe epilepsy [12,20]. More studies are needed to draw conclusions on the potential effects of clock genes in homogeneous samples of epilepsy patients.

**Table 1:** An overview of circadian molecular system and epilepsy studies.

Authors	Study Model	Alterations of Molecular Circadian System	Tissues Examined	Alterations of Sleep–Wake and Circadian Markers
Liang et al., 2024 [9]	Pilocarpine-induced SE mouse model.	*Bmal1*, *Clock*, *Cry1*, *Cry2* ↓ expression, *Per1* and *Per2* ↑ expression.	SCN and hippocampus.	Lower core body temperature oscillations.
Wallace et al., 2018 [13]	*Kcna1* knockout mouse model.	*Clock*, *Per1* and *Per2* ↓ expression.	Anterior hypothalamus.	Arrhythmic rest–activity patterns
Matos et al., 2018 [8]	Pilocarpine induced SE rat model.	Phase advance of *Bmal1* acrophase in epileptic condition; lack of rhythmicity of *Cry1* and *Cry2* expression in post-SE condition; lack of rhythmicity of *Per1*, *Per2* expression in post-SE and epileptic condition, and *Per3* expression in epileptic condition.	Hippocampus.	Intact circadian rhythm of rest–activity; higher activity in both light and dark phases; higher intracycle variability.
Gerstner et al., 2014 [10]	Electroshock mouse model.	*Bmal1* expression unaffected by seizures.	Hippocampus.	No diurnal profile of seizures in *Bmal1* knockout mice compared to WT.
Zhang et al., 2021 [12]	KA-induced acute and pilocarpine-induced chronic seizure mouse models.	*Rev-erbα ↑* expression and *Bmal1*, *Clock*, *E4bp*, and *Dbp* expression ↓ in KA model.	Hippocampus, cortex in KA model mice.	Seizure reduction in *Rev-erbα* knockout mice in both KA and pilocarpine models.
Rocha et al., 2017 [16]	Pilocarpine-induced SE model.	MT1 mRNA expression ↑ 2 and 11 h post-SE; MT2 mRNA expression ↑ 5 and 11 h post-SE; MT2 mRNA expression ↓ in the silent phase.	Hippocampus.	-
Yamakawa et al., 2023 [14]	GEARS model.	Dysregulation of *Per1*, *Cry1* and *Clock*, *Bmal1*.	Hypothalamus, hippocampus, liver, small intestine.	-
Yamakawa et al., 2023 [14]	KA-induced SE model.	Dysregulation of *Cry1*, *Clock*, and *Bmal1*.	Hypothalamus, liver.	-
Yamakawa et al., 2023 [14]	KA-induced SE model.	Dysregulation of *Cry1* and *Clock*.	Hippocampus.	-

Abbreviations: SE—status epilepticus; *Clock*—circadian locomotor output cycles kaput; *Bmal1*—Basic helix-loop-helix ARNT-like protein 1; *Per*—period; *Cry*—Cryptochrome Circadian Regulator 1; *Rev-erbα*—nuclear receptor subfamily 1 group D member 1; *Dbp*—D-Box Binding PAR BZIP Transcription Factor; MT1 and MT2—melatonin receptors 1 and 2, respectively; SCN—suprachiasmatic nucleus; *Kcna1*—Potassium Voltage-Gated Channel Subfamily A Member 1; KA—kainic acid; GEARS—Genetic absence epilepsy rat from Strasbourg, WT—wild type, ↓—decrease; ↑—increase.

## 4. Effects of Seizures and Interictal Activity on Sleep and Wakefulness and Circadian Rhythm

An increasing body of evidence shows that seizures and interictal activity have circadian and even circannual properties [21,22]. Seizure forecasting is a term describing the prediction of seizure occurrence and is important for managing the timed use of antiseizure medications, a process called chronotherapy [22]. Some epilepsy syndromes have more predictable ictal and interictal activities in relation to vigilance states, e.g., seizures in genetic generalized epilepsies tend to happen upon awakening, while the majority of seizures in self-limiting focal epilepsies occur during sleep [23]. However, seizures and, at least to some extent, interictal activity are events that can significantly affect a person’s quality of life, but data on whether and how ictal and interictal activity affects sleep–wake rhythms are quite scarce. Animal studies have shown that pentylenetetrazol-induced generalized seizures are associated with increased wakefulness before the onset of seizures and diminished slow-wave and REM sleep [24]. To our knowledge, there are no human studies on pre-ictal effects on sleep. A prospective case–control study of 200 epilepsy patients and 100 healthy controls revealed that non-seizure-free patients experienced significantly increased excessive daytime sleepiness (EDS) compared to seizure-free patients. Moreover, 41% of patients with epilepsy complained that nocturnal seizures resulted in EDS the following day, 16% required a nap after a generalized seizure, and 5% had a disturbed night of sleep following a generalized seizure [25]. Similarly, another prospective case–control study that included 160 people with epilepsy showed a significant positive correlation between seizure frequency, increased EDS, and worse sleep quality measures on the Pittsburg Sleep Quality Index (PSQI) [26]. It remains unclear how the much severity and frequency of different types of seizures affect a person’s rest–activity rhythms.

Changes in sleep architecture have been studied extensively in people with epilepsy. An overview of relevant studies is provided in Table 2.

Ictal and interictal activity are associated with increased awakening and arousal, with symptomatic seizures being more frequently associated with awakening [27]. The most common, although to a different extent, findings regarding the disruption of sleep parameters in both mixed and homogenous epilepsy samples are a reduced sleep efficiency and total sleep time, prominent sleep fragmentation, and a reduction in slow-wave and REM sleep [27,28,29,30,31,32,33,34,35,36,37,38,39,40,41,42,43,44]. It is more likely that people with drug-resistant epilepsy and seizures during the night will have their sleep continuity disrupted more severely. Some studies have found that sleep-disordered breathing could be more common in people with epilepsy [34,37], although this could be an accidental finding in populations with fewer comorbidities.

**Table 2:** Changes in sleep structure in epilepsy patients.

Authors	Epilepsy	Alterations of Sleep Architecture
Peter-Derex et al., 2020 [27] Pereira et al., 2012 [30] Arhan et al., 2021 [36] Zanzmera et al., 2012 [41] Yeh at al., 2021 [42]	Treatment-resistant epilepsy	WASO ↑, AI ↑, SE ↓, TST ↓, %REM ↓, %N3 ↓, REM latenc y↑
Calvello et al., 2023 [28] Arhan et al., 2021 [36]	Drug-naïve FE and GE	SE ↓ in FE and GE [28] Sleep stage shift ↑, awakenings ↑, WASO ↑, %N2 ↑ in FE [28] Sleep onset latency ↑, REM latency ↑, %N1 ↑, awakenings ↑ in FE [36]
Mekky et al., 2017 [29] Roshan et al., 2017 [35] Krishnan et al., 2014 [44]	JME	%wake ↑, WASO ↑, SE ↓, sleep onset latency ↑ [29,35,44] REM latency and duration ↑, AI ↑, AI associated with disease duration and age of seizure onset, PLMI ↑ [29] %REM ↓, %N1 ↑ [35]
Maganti et al., 2005 [32] Yadav et al., 2021 [37] Hamdy et al., 2020 [38]	Idiopathic GE	%N1 ↑, REM latency ↑ [32] REM duration ↓, AHI ↑, arousals ↑, % of oxygen desaturation ↑ [37] ↓ %REM and REM latency ↑ in drug-naïve patients [38]
Nakamura et al., 2017 [31]	TLE	↓ %REM in patients with left TLE
Gogou et al., 2017 [34] Bruni et al., 2010 [43]	Self-limited epilepsy with centrotemporal spikes (Rolandic)	↓ %REM, OAI and OAHI ↑ [34] REM latency ↑, SE ↓, TST ↓, reduced CAP rates [43]
Hadar et al., 2024 [33]	FE	%N3 ↓ overall %N3 ↑, %REM ↑, %N2 ↓ in FE with generalization of seizures compared to FE without generalization
Wang et al., 2024 [39] Shaheen et al., 2012 [40]	Mixed samples of epilepsy	SE ↓, TST ↓, %REM ↓, %N3 ↓, AI ↑, oxygen desaturation index ↑ in elderly patients with epilepsy [39] Greater frequency of seizures in OSA patients [40]

Abbreviations: REM—rapid eye movement sleep; CAP—cyclic alternating pattern; TST—total sleep time; SE—sleep efficiency; WASO—wake after sleep onset; N1, N2, N3—1, 2 and 3 stages of NREM sleep, respectively, AI—arousal index; PLMI—periodic limb movement index; OAI—obstructive apnea index; OAHI—obstructive apnea-hypopnea index; AHI—apnea–hypopnea index; OSA—obstructive sleep apnea; GE—generalized epilepsy; FE—focal epilepsy; TLE—temporal lobe epilepsy; JME—juvenile myoclonic epilepsy; ↓—decrease; ↑—increase.

People with epilepsy may have different sleep–wake profiles based on their age and type of epilepsy. A recent systematic literature review that included 11 studies showed that sleep–wake preferences varies among children with epilepsy, while in adults, waking up early in the morning was preferred, and going to sleep later in the evening was more dominant in patients with generalized epilepsies [45]. Most of these studies are questionnaire-based, and only two involved testing a circadian rhythm biomarker dim-light melatonin onset [45]. It was shown that circadian system impairment in epilepsy might not be related to seizures [13]; however, there are some controversial results in human studies. Decreased melatonin levels in the morning hours were a common finding in patients with febrile seizures [46]. Also, increased melatonin levels were found early in the postictal state, but the base levels were decreased hours after a seizure [47,48]. Further, morning serum melatonin levels were decreased in patients with continuous spikes and waves during sleep, and the basal serum levels of melatonin also decreased in electric status epilepticus in sleep (ESES) patients [49,50]. Despite evidence of an increase in melatonin receptors after a prolonged seizure in previously mentioned rodent studies, no circadian melatonin profile alterations were observed in a small study of epilepsy patients experiencing seizures [51]. Cortisol is another hormone that has circadian properties, with peak levels occurring within the first hour of awakening and being influenced by a variety of factors, such as cognition, mood, or light levels [52]. Seizures can influence cortisol secretion; in particular, cortisol serum levels were found to be increased in the first hour after a seizure [53]. Interestingly, both ictal and interictal activity have been found to be correlated with circadian changes in cortisol levels [53,54]. Peak seizure occurrence follows an increase in cortisol in the early hours of the morning, with a time delay of approximately 2 h. However, another seizure peak in the afternoon was found to be independent of cortisol rhythms [55]. There is a lack of literature regarding other circadian biomarkers, such as core body temperature, and their association with seizures and interictal activity. However, current data on melatonin and cortisol alterations in relation to seizures and prominent interictal activity show the disturbance of normal rhythms in people with epilepsy.

## 5. Sleep Disturbances in Different Epilepsy Etiologies

Epilepsy is a heterogeneous disorder. Based on the position of the International League Against Epilepsy Commission for Classification and Terminology Comorbidities, epilepsy can be classified according to its etiology, which includes metabolic, structural, genetic, immune, infectious, and unknown etiologies. Some etiologies might overlap, e.g., structural and genetic or genetic and metabolic [56]. In this paper, we review a few selected syndromes, of which epilepsy is one of the most debilitating phenomena, with sleep problems being a frequent comorbidity. The sleep problems observed in specific syndromes related to epilepsy are summarized in Table 3.

### 5.1. Chromosomal Abnormalities

Angelman syndrome is a neurodevelopmental disorder caused by the loss of function in the maternal copy of ubiquitin-protein ligase E3A on chromosome 15q11-13 and can be a cause of severe epilepsy, although the genotype–phenotype correlation may vary [84]. The majority of Angelman syndrome patients experience sleep issues due to the dysregulation of both the circadian rhythm (process C) and sleep homeostasis (process S), resulting in the abnormal secretion of melatonin, difficulties falling asleep, frequent awakening at night, and sleep deprivation [57,58].

People with Down syndrome (21 chromosome trisomy) tend to have a variety of sleep problems, such as obstructive sleep apnea (OSA), difficulties initiating and maintaining sleep, and parasomnias [59,60,61]; however, some evidence of circadian disturbances has recently emerged. Adolescents with 21 trisomies lack the typical shift to a more delayed sleep phase; meanwhile, a decrease in interdaily stability and an increase in intradaily variability—altered circadian rhythmicity markers—was seen in adults with 21 trisomies [62].

### 5.2. Gene Abnormalities

Fragile X syndrome patients have a relatively high prevalence of sleep disturbances, including problems initiating and maintaining sleep, bruxism, and snoring [63]. In a small sample of fragile X syndrome patients, dim light melatonin onset peak was found to be around noon and not in the evening hours. Further, actigraphy studies showed low interdaily stability and high intradaily variability [64]. However, larger studies are needed to replicate these results.

Changes in the MECP2 gene cause Rett syndrome, which poses a high risk of recurrent seizures. Consensus guidelines on managing Rett syndrome across one’s lifespan include sleep problems as one of the areas of multidisciplinary care [85]. Therefore, being suspicious of sleep-disordered breathing as well as insomnia is advised [65,67,68]. Among sleep disorders, irregular sleep–wake patterns were found in a questionnaire study of the patient’s parents [86]. *FOXG1* syndrome and *CDKL5* deficiency disorder have overlapping features with Rett syndrome; these features include epilepsy, developmental delay, hyperkinetic movements, and feeding difficulties. Patients with *FOXG1* syndrome and *CDKL5* deficiency disorder tend to have a short sleep duration and frequent awakenings at night [66,69].

Tuberous sclerosis complex (TSC) is a rare neurocutaneous disorder caused by changes in the *TSC1* and *TSC2* genes and is characterized by hamartomas that affect multiple organ systems due to the reduced inhibition of the mTOR pathway. An altered sleep–wake rhythm and sleep microstructure in TSC are associated with changes in the mTOR pathway, altered core clock gene expression, and the increased expression of orexin [19,87,88]. In a study of 177 children with TSC, the most prevalent sleep problems were disorders related to sleep–wake transitions, difficulties initiating and maintaining sleep, and sleep-disordered breathing; however, disturbed sleep was more related to comorbid neuropsychiatric disorders than nocturnal seizures or antiseizure medication. Nevertheless, a higher prevalence of sleep disorders was observed in children with both TSC and epilepsy [70].

Changes in the *SCN1A* gene are the main factor causing Dravet syndrome—a developmental and epileptic encephalopathy associated with treatment-resistant seizures. A long-term corticography study in a rodent model of Dravet syndrome revealed impaired circadian regulation, the particularly high intradaily variability of NREM sleep unrelated to interictal activity, and an elongated circadian period of sleep in conditions of constant darkness [89]. Up to three quarters of Dravet patients experience sleep problems, including difficulties initiating and maintaining sleep, sleep–wake transition disorders, and sleep-disordered breathing [71,72]. However, it is also reported that nocturnal seizures significantly contribute to awakenings [72]. Meanwhile, antiseizure medications are related to restlessness and drowsiness [72].

Changes in the *SLC2A1* gene are the primary cause of GLUT1 transporter deficiency, which is characterized by a variety of neurological symptoms, including seizures, movement disorders and developmental delay, and is primarily treated with a ketogenic diet. Although not extensively, some sleep problems, such as excessive daytime sleepiness, have been reported to respond to treatment with a ketogenic diet [73].

Defects in the *ZEB2* gene are the main etiological factor in Mowat–Wilson syndrome, which is characterized by dysmorphic facial features, epilepsy, neurodevelopmental problems, musculoskeletal abnormalities, congenital cardiovascular problems, visual problems, and urogenital and bowel anomalies [90]. Patients with Mowat–Wilson syndrome tend to suffer from sleep–wake transition disturbances and difficulties initiating and maintaining sleep. It has also been found that their total sleep time and deep sleep time are reduced, and that their arousal index is increased in polysomnographic studies [74,75].

### 5.3. Structural Lesions

Hippocampal sclerosis is a structural lesion within the hippocampus of the temporal lobe that is characterized by gliosis and the loss of hippocampal neurons; it is usually an abnormal finding in patients with drug-resistant mesial temporal lobe epilepsy and aging individuals, and as a dual pathology in other disorders such as Sturge–Weber syndrome [91,92]. Due to medication-resistant seizures, patients with temporal lobe epilepsy and hippocampal sclerosis often require epilepsy surgery. Few studies have examined the sleep profiles of these patients before and after surgical treatment. Yarangula and colleagues found lower PSQI scores, a longer sleep duration, and less deep sleep compared to healthy controls. However, a post-operative sleep questionnaire assessment did not show improvement [76]. Another prospective study that included both polysomnography studies in both the pre-surgery and post-surgical periods showed significantly reduced sleep instability via cyclic alternating pattern measurements in the post-surgical period [77].

Hypothalamic hamartoma is a lesion located in the ventral hypothalamus and is associated with treatment-resistant epilepsy and endocrine anomalies such as precocious puberty [93]. Many circuits involving sleep and wakefulness are in the hypothalamus; however, very few cases of sleep disturbance and hypothalamic hamartoma have been reported. Tezer and colleagues reported a 25-year-old female patient with secondary hypersomnia and gelastic seizures whose hypersomnolence improved after initiating modafinil [78].

### 5.4. Metabolic Disorders

Mitochondrial disorders are a broad group of disorders characterized by multiorgan involvement with a substantial impact on the nervous system, presenting with seizures of varying severity, stroke-like episodes, or visual impairment [94]. Sleep-disordered breathing, including obstructive sleep apnea, central sleep apnea, and sleep hypoventilation, is the most prevalent sleep disturbance in both children and adults with mitochondrial disorders causing EDS [79,80]. Myopathic and peripheral neuropathic disturbances are seen in mitochondrial disorders resulting in muscle and bulbar weakness, and are the main factor causing sleep-disordered breathing in this population [94].

### 5.5. Autoimmune Disorders 

LGI1 (leucine-rich, glioma-inactivated 1 protein) and CASPR2 (contactin-associated protein 2) antibody encephalitis have a broad spectrum of phenotypes; however, common symptoms include seizures (including faciobrachial dystonic seizures in LGI1-antibody encephalitis), cognitive disturbances, dysautonomia and hyponatremia [95]. LGI1/CASPR2 antibody-positive patients demonstrate symptoms of insomnia, sleep-disordered breathing, and prominent features of REM-behavior disorder [81].

Anti-NMDAR (N-methyl-D-aspartate receptor) encephalitis has acute or subacute onset, with prominent psychopathological symptoms, movement disorders, seizures and dysautonomic features [81]. Sleep problems tend to shift during the course of the disease, with pronounced insomnia at the onset and hypersomnia after the acute stage; patients can also develop both REM and NREM parasomnias [82,83].

## 6. Comorbidities

Comorbidities in epilepsy, both somatic and psychiatric, are a frequent phenomenon with a considerable burden [96]. The associations between epilepsy and comorbidities can be causative; these include, for example, frequent seizures and cognitive decline in epileptic encephalopathies. These associations can also be direct or indirect when there is an intermediate factor causing a comorbidity, such as treatment with antiseizure medication resulting in side effects. Some comorbidities are due to shared risk factors like a genetic etiology [97]. Addressing all comorbidities in epilepsy is beyond the scope of this review. However, some are particularly associated with sleep problems.

### 6.1. Mood Disorders, Psychosis and Anxiety

A series of papers, including guidelines for the treatment of depression and anxiety in people with epilepsy, published by the International League Against Epilepsy in the past two decades have highlighted the importance of being aware of psychiatric comorbidities [98,99,100]. Neurobiology involving psychiatric comorbidities overlaps with epilepsy and can have temporal relationships with seizures or exist as separate conditions, although there is sometimes an atypical pattern in comparison to people without epilepsy [99]. Sleep problems in psychiatric disorders are a subject of research as the two usually coexist with sleep problems, usually preceding mood and other psychiatric disorders. It is hypothesized that stress in early life leads to alterations in one’s reactions to further stress and contributes to changes in brain organization, including sleep regulation, which in turn increases the risk of mood disorders in the future [101]. Insomnia, loss of sleep, and circadian rhythm alterations in people with mood disorders cause the accumulation of toxic proteins, neuroinflammation, oxidative stress, and reduced neuroprotection, ultimately leading to continuous neuronal damage and progression in mood disorders [102]. In a recent systematic review and meta-analysis, Scott and colleagues showed that prior sleep problems, particularly insomnia, increase the likelihood of mood disorders or psychosis in adolescence and early adulthood [103]. Similarly, any sleep problems pose a risk of depression in old age [104]. Sleep problems, including the subjective perception of sleep quality and difficulties in and maintaining sleep, are described in up to half of patients with both acute and chronic psychosis; patients at risk of psychosis episodes also tend to report sleep disruptions [105]. The evening chronotype was also found to be associated with schizophrenia and major depressive disorder [106]. Anxiety disorders are mostly related to insomnia and have a reciprocal relationship in a mutually reinforcing manner [107]. Additional risk factors for anxiety, such as polytherapy and psychiatric antiseizure medication side effects, were found in people with epilepsy [108]. In the consensus-based recommendations for the diagnosis and treatment of anxiety and depression in children and adolescents with epilepsy, it is highlighted that sleep monitoring is required as sleep deterioration tends to worsen the course of anxiety and depression [100].

### 6.2. Neurodevelopmental Disorders

Sleep problems, particularly insomnia, are commonly complained of by caregivers of people with autism spectrum disorder (ASD), and are most likely multifactorial [109]. Circadian clock gene polymorphisms, the diminished biosynthesis of melatonin, arousal and sensory dysregulation, as well as behavioral issues are the main driving factors [110,111,112]. Apart from insomnia, children and adolescents with ASD tend to have more frequent parasomnias, sleep-disordered breathing, and EDS. Similarly, adults with ASD are reported to have more frequent EDS [113,114]. Although behavioral therapies are one of the mainstay treatments for sleep problems in ASD, a subset of unresponsive patients need additional therapies. A randomized double-blind controlled trial included 125 pediatric patients, of whom 96.8% had ASD and showed a significantly improved total sleep time and decreased sleep latency when given 2–5 mg of prolonged-release melatonin [115].

A variety of sleep difficulties are prevalent in attention deficit hyperactivity disorder (ADHD). Miano and colleagues described different sleep phenotypes in ADHD patients, including EDS (or narcolepsy-like), sleep onset delay insomnia, obstructive sleep apnea, restless leg syndrome/periodic limb movement, and the EEG interictal discharges phenotype [116,117]. The results of studies on the sleep micro- and macrostructure in people with ADHD seem controversial. Overall, the macrostructure of sleep in ADHD patients seems to be unaltered; however, an increase in the slow sleep spindle amplitude duration and frequency, a lower cyclic alternating pattern rate in NREM sleep, and an increased cyclic alternating pattern rate in deep sleep in patients with the SDB phenotype were seen in some studies [118,119,120,121,122,123]. A substantial number of people with ADHD have interictal discharges, which might be associated with higher attention deficit and impulsive traits as well as poorer cognitive abilities, although more studies are needed to confirm such results [124,125].

## 7. Antiseizure Medications

Antiseizure medications (ASMs) have a broad spectrum of effects on the central nervous system; in particular, sedation is of concern when discussing the effects of ASMs on sleep and wakefulness. Importantly, a subset of patients with monotherapy will not achieve seizure freedom and will require more than one ASM with overall negative outcomes regarding quality of life [126]. We further discuss the effects of ASMs that are reported to exhibit effects on sleep, wakefulness, and sleep structure. The effects of ASMs on sleep are summarized in Table 4.

### 7.1. Activators of GABA_A_ Receptors

Benzodiazepines (BZDs) are a group of medications widely used for the treatment of a variety of epilepsy syndromes, either as a long-term treatment or as rescue medication for prolonged seizures. Benzodiazepines act as modulators of GABA_A_ receptors, causing a dose-dependent sedating effect; in particular, interaction with the high-affinity α–γ subunit interface is associated with seizure-inhibiting action [153]. Due to their mechanism of action, BZDs, among other ASMs, are known to have profound effects on sleep and wakefulness. The chronic use of BZDs may cause alterations in the macro- and microstructure of sleep, such as an increase in REM sleep latency and the percentage of stage 1 NREM sleep, and a significant reduction in the rate of cyclic alternating patterns, indicating reduced arousability [127]. Meanwhile, EDS is a frequently observed side-effect of BZDs that can impair daytime functioning [128]. Interestingly, increased episodes of NREM parasomnias, such as sleepwalking, are also reported for long-term BZD users, even though this group of medications is used for the short-term treatment of parasomnias [129,154]. The long-term use of BZDs is also associated with cognitive decline, particularly in sustained attention and processing speed domains [155]. Similarly, patients with mild cognitive decline show longer N1 sleep and less REM sleep; however, whether alterations in the sleep structure of patients with long-term BZD treatment could reflect altered cognition still remains to be seen [156].

Phenobarbital (PB) is a first-generation ASM that enables the opening of chloride ion channel properties via the GABA_A_ receptor [157]. PB is sometimes used in clinical practice for long-term therapy and, more commonly, for the acute treatment of SE patients and neonates presenting with seizures. EDS is one of the most common side effects of PB [130,131]. PB has been shown to reduce sleep latency, arousability, and REM sleep, as well as increase light sleep [132].

### 7.2. ASMs with Multiple Mechanisms of Action

Valproic acid (VPA) is an old ASM that has a profound impact on the GABA-ergic system via multiple mechanisms of action [158]. A study of 60 patients with epilepsy showed a significant reduction in REM and an increase in N1 sleep and arousal 3 months into valproate administration [133]. Similar to opioids, VPA has shown patterns of central sleep apnea in polysomnographic studies in a few reported cases [134]. In practice, some patients treated with VPA report increased drowsiness. Indeed, a longer total sleep duration is seen in patients with long-term VPA treatment [135].

Highly purified cannabidiol (CBD) exhibits a broad spectrum of targets: G protein-coupled receptor 55, transient receptor potential vanilloid 1, equilibrative nucleoside transporter 1 are significant for seizure inhibition; meanwhile, the endocanabinoid system, particularly CB1 and CB2 receptors, demonstrate sleep-promoting properties [159]. Somnolence was reported to be one of the most common adverse effects of CBD in patients with treatment-resistant epilepsy syndromes, with a prevalence ranging from 16 to 29% [137,138,139]. CBD was shown to increase slow-wave and REM sleep in patients with insomnia without increasing the total sleep time [136]. Further, no effect on sleep parameters was seen in polysomnographic studies in healthy volunteers in the CBD versus placebo group [160]. CBD is usually prescribed in treatment-resistant epilepsies such as Lennox–Gastaut, Dravet syndromes, and tuberous sclerosis complex—all of which are characterized by ASM polytherapy; in particular, clobazam (CLB) is known to increase levels of CBD metabolites [161]. However, studies of CBD drug–drug interactions are scarce, and it is not yet safe to rule out the involvement of polytherapy in the EDS exhibited by CBD users.

### 7.3. Sodium Channel Blockers

Carbamazepine (CBZ) is one the oldest voltage-gated sodium channel blockers. In a study of 40 patients with temporal lobe epilepsy and 40 healthy controls, treatment with CBZ did not lead to increased EDS. However, a poorer subjective sleep quality and a disruption of the sleep microstructure via an increase in cyclic alternating pattern rates were reported [140]. Conversely, the chemically related sodium channel blocker oxcarbazepine (OXZ) was reported to increase the total sleep time. However, this was compared to patients treated with levetiracetam (LEV) in both a cross-sectional and longitudinal study that included a total of 120 patients [141]. Another sodium channel blocker, lamotrigine (LTG), is reported, although not consistently, to increase the REM sleep percentage, decrease slow-wave sleep, and increase stage 2 NREM sleep [142,143]. In a sample of 109 epilepsy patients, the initiation of LTG was associated with insomnia requiring discontinuation or dose reduction in seven patients [162]. Some controversial results were reported with the newer third-generation sodium channel blocker lacosamide (LCZ). One study showed increased sleep efficiency and a decreased N1 and N3 sleep percentage in patients with focal epilepsy, although such results were achieved along with complete seizure control [144]. Moreover, a study of 25 healthy volunteers showed no significant effects on sleep parameters after 22 days of lacosamide administration, indicating that the improvement of sleep parameters is more likely to be related to diminished ictal and interictal activities [163]. No associations between LCZ and EDS were found in a study of 52 adults with focal epilepsy, although improvements in subjective sleep measures were observed [164].

### 7.4. Inhibitors of Voltage-Gated Calcium Channel α2δ Subunits (Gabapentinoids)

Gabapentin (GBP) and pregabalin (PGB) are widely used in sleep medicine, primarily as one of the treatment options for restless legs syndrome [165]. A randomized, double-blind, placebo-controlled, multicenter trial revealed the significant effects of a 250 mg dose of GBP, including a reduction in awakenings after sleep onset and in the percentage of N1 sleep, and an increase in REM sleep both at day 1 and 28 of GBP exposure in patients with occasional sleep disturbances [145]. Similarly, PGB is associated with an increase in slow-wave sleep and reduced sleep-onset latency, as well as better sleep efficiency [147]. A systematic review and meta-analysis of a large sample of patients with neuropathic pain showed that gabapentinoids induce EDS, albeit with improved night sleep [146]. Similar doses of gabapentinoids are administered for patients with focal seizures. Therefore, EDS is expected in patients taking higher doses of GBP or PGB to achieve seizure control.

### 7.5. Synaptic Vesicle Protein 2A Modulators

LEV is a second-generation ASM that acts as a synaptic vesicle protein 2A modulator, thus diminishing epileptogenesis [166]. Increased night-time awakenings without the disruption of other sleep parameters in a small sample of healthy participants was found to be associated with 1 month of LEV therapy [148]. Similarly, a study of 29 epilepsy patients with LEV monotherapy revealed increased arousals and awakenings, as well as an increase in N2 and a decrease in N3 sleep after three months of LEV use [149]. On the contrary, a study of 14 healthy adults did not show an increased arousal index. Moreover, significant increases in the total sleep time, sleep efficiency and deep sleep were observed, as well as a decreased percentage of REM sleep [150]. Another study of focal epilepsy patients also showed a decreased percentage of REM sleep and increased subjective somnolence after 7 days of LEV therapy. However, no changes in multiple sleep latency tests were observed [151]. Overall, the results of sleep studies of patients with LEV monotherapy seem to be controversial, although low sample sizes, differences in methodology, and patient populations play a significant role. Interestingly, mood changes—the most prominent side effect of LEV—were associated with the early chronotype in people with epilepsy, and as suggested by the authors of the study, these findings might be related to multiple factors, including the internalization of depression symptoms in evening chronotypes and genetic factors [167].

### 7.6. AMPA A-Type Glutamate Receptor Inhibitors

Perampanel (PER) is a third-generation ASM that diminishes the excitatory effects of glutamate via α-amino-3-hydroxy-5-methyl-4-isoxazolepropionic acid (AMPA) receptor inhibition [168]. In a study of 17 treatment-resistant epilepsy patients, PER was shown to improve sleep parameters, such as the total sleep time and sleep efficiency, and increase slow-wave sleep, although a significant improvement in seizures was seen in most of the patients, highlighting the expected impact of seizure control on sleep [152]. A study of 10 patients with treatment-resistant focal epilepsy showed no effect of PER on their subjective sleep quality and EDS, as well as their objective actigraphy and mean wakefulness test results [169]. Another study showed significant seizure reduction and reduced mean sleep latency during daytime in patients treated with add-on PER [170]. Overall, sleep studies of patients treated with PER are scarce. However, some sleep parameters tend to improve via the control of seizures.

## 8. Discussion

In this review, we have adopted a rather pragmatic approach to sleep and wakefulness alterations in relation to epilepsy. We discussed how the sleep–wake rhythm, although to different extents, can be impacted by virtually every domain of epilepsy, including by molecular and etiological factors, comorbidities, seizures, interictal activity and antiseizure medication.

In our review, we have included animal studies of molecular circadian systems due to a lack of studies with humans. However, a growing body of evidence shows that alterations in the molecular circadian feedback loop system have a profound negative impact on metabolic and other domains of health. The misalignment of external cues to the circadian system is associated with an increased body weight, insulin resistance, and dyslipidemia [171]. Indeed, at least half of people with epilepsy have metabolic syndrome, although this is related to multiple factors, such as antiseizure medications and a lack of physical activity [172]. Moreover, alterations in *CLOCK* gene expression and thus changes in the dopaminergic, GABAergic, and glutamatergic systems are associated with known epilepsy comorbidities, such as mood disorders, ADHD, autism spectrum disorder, and schizophrenia [173]. So far, different epilepsy experimental models exhibit different clock gene characteristics in the central nervous system and peripheral tissues. Whether these changes have a meaningful clinical impact on sleep–wake and metabolic rhythms, particularly in people with epilepsy, remains to be seen. However, this prompts further research into more homogeneous samples of epilepsy participants. Also, more human studies are needed to confirm the results of studies utilizing animal experimental models.

So far, it is difficult to quantify how seizures and interictal activity affect sleep–wake rhythms. As shown previously, seizures during sleep and interictal activity are related to increased arousability and sleep fragmentation. Continuous fragmented sleep has a negative impact on cognition and increases the risk of obesity and congestive heart failure, among other deleterious effects [174,175,176]. The postictal state also has a myriad of manifestations, including drowsiness, cognitive dysfunction, and changes in mood, with some even lasting days [177]. Both seizures and related behavioral aspects might play a role in the disruption of normal sleep–wake rhythms. It is interesting that even seizure-free patients tend to demonstrate disturbed sleep–wake rhythms, particularly a delayed sleep–wake pattern, less regular activity–rest patterns, and a fragmented sleep–wake cycle [178]. The impairment of sleep–wake rhythms is most likely multifactorial. Yet, changes in circadian biomarkers, such as melatonin and cortisol, in relations to seizures and interictal activity are seen in people with epilepsy.

Comorbid conditions, syndromes associated with epilepsy, and antiseizure medications by themselves are associated with alterations in sleep profiles via different mechanisms, and it is logical to assume that these factors usually work in combination. Therefore, some sleep issues or alterations might always be present in a patient with epilepsy.

Some disorders present with a phenotype that involves both seizures and sleep disturbances, which might be unrelated, as in cases of mitochondropathies, where excessive daytime sleepiness is primarily due to sleep-disordered breathing. Meanwhile, other syndromes involving nighttime seizures, such as tuberous sclerosis complex, could severely impact sleep due to frequent awakenings. Most of the disorders described in this review are considered rare; therefore, sleep studies are quite scarce. However, patients with rare disorders are usually subjected to multidisciplinary care in specialized centers with an increased awareness of such issues.

Psychiatric comorbidities are not uncommon [179]. Insomnia is a common symptom of psychosis, depression, and anxiety disorders. Similarly, a variety of sleep problems are prevalent in neurodevelopmental disorders, such as autism spectrum disorder and ADHD. Some forms of epilepsy are particularly sensitive to sleep deprivation. Idiopathic (genetic) generalized epilepsies, especially juvenile myoclonic epilepsy, which is also known as awakening epilepsy, are characterized by enhanced interictal activity and an increased risk of seizures after sleep deprivation [23]. Moreover, in cases of focal epilepsy, an increase in sleep duration is associated with a lower risk of seizures in the next two days [180]. Discussing psychiatric and neurodevelopmental comorbidities in people with epilepsy is important, as improving sleep-related issues during a patient‘s visit would be beneficial to both their quality of life and seizure control.

Another layer of complexity in the issues discussed is introduced with ASM. As was shown previously, almost every ASM has some effect on sleep and wakefulness. The most common side effect is EDS, which is mostly seen in BZD and barbiturate users. So far, it is difficult to quantify the burden of sleep structure alterations associated with the chronic use of ASM. This is due to studies including low sample sizes and heterogeneous groups of patients, with the most controversial results presented in CBD, LTG, LCZ, LEV, and PER studies. Some ASM, such as PER, are shown to improve sleep structure, but this is most likely related to the control of seizures.

Currently, the management of sleep disorders and sleep problems in people with epilepsy remains similar to that without epilepsy. However, some additional aspects should be considered. Sleep hygiene is related to a worse quality of life in people with epilepsy [181], therefore suggesting that proper sleep practices, including regular sleep schedules, are essential. An expert opinion on managing sleep disturbances in people with epilepsy has recently been published. The authors suggest that, along with proper sleep practices, the revision and optimization of antiseizure medications, the consideration of nocturnal seizures, and the detection and treatment of concomitant sleep disorders based on currently existing guidelines are essential if the patient complains of insomnia or excessive daytime sleepiness [182].

## 9. Future Directions

Studies exploring the molecular circadian system in human epilepsy patients are incredibly scarce. Animal studies show various alterations in molecular circadian systems in different epilepsy models. Future studies on molecular circadian systems should include homogeneous samples of epilepsy patients. Moreover, data on peripheral circadian markers and sleep–wake rhythm behaviors in people with epilepsy are still needed. Taking together, these data could provide valuable insights into the different circadian profiles of epilepsy patients, circadian aspects of seizures, and interictal activity, and would become a useful tool for predicting seizures and applying pharmacological treatment.

Sleep disturbances are common in various syndromes and comorbidities associated with epilepsy. Various sleep problems are associated with syndromes related to epilepsy. However, these syndromes are usually considered rare disorders. Therefore, multicenter studies that include larger sample sizes of patients are needed. Further, studies of epilepsy patients with comorbid psychiatric and neurodevelopmental disorders could highlight the differences between populations with and without seizures.

Sleep studies exploring the effects of epilepsy on sleep include mixed samples of patients, such as drug-naïve patients and patients with polytherapy. To better distinguish alterations in sleep parameters in people with epilepsy, more homogenous samples of drug-naïve patients should be included in longitudinal studies measuring polygraphic parameters before and during treatment.

## 10. Conclusions

In this review, we have presented multiple epilepsy-related factors that impact sleep and wakefulness. The molecular circadian system is likely involved in both ictal activities and sleep–wake rhythm disturbances. However, more studies with human epilepsy patients are needed. Seizures and interictal activity could be related to sleep and circadian rhythm disruption. Therefore, adequate seizure control is required. Comorbid conditions and syndromes by themselves have a negative impact on sleep and wakefulness; thus, screening for sleep problems in these conditions is mandatory. Alterations in the sleep structure and excessive daytime sleepiness can be related to antiseizure medications; however, bigger longitudinal studies are needed to confirm the current findings.

## Figures and Tables

**Table 3:** Syndromes associated with epilepsy and sleep disturbances.

Authors	Syndrome	Sleep Problems and Disorders
Takaesu et al., 2012 [57] Ehlen et al., 2015 [58]	Angelman syndrome	Circadian rhythm dysregulation; Disruption of sleep homeostasis.
Hoffmire et al., 2014 [59] Carter et al., 2009 [60] Lin et al., 2014 [61] Lovos et al., 2021 [62]	21 chromosome trisomy	Insomnia; OSA; Parasomnias; Circadian rhythm dysregulation.
Budimirovic et al., 2022 [63] Dueck et al., 2020 [64]	Fragile X syndrome	Insomnia; Bruxism; Snoring; Possible circadian rhythm dysregulation.
Wong et al., 2015 [65] Mangatt et al., 2016 [66] Sarber et al., 2019 [67] Zhang et al., 2022 [68] Wong et al., 2023 [69]	Rett and Rett-like syndromes	Insomnia; Laughing and night screaming; Sleep-disordered breathing.
Moavero et al., 2022 [70]	Tuberous sclerosis complex	Sleep–wake transition disorders; Insomnia; Sleep-disordered breathing.
Licheni et al., 2018 [71] Van Nuland et al., 2021 [72]	Dravet syndrome	Sleep–wake transition disorders; Insomnia; Sleep-disordered breathing.
De Giorgis et al., 2016 [73]	GLUT1 transporter deficiency	Excessive daytime sleepiness.
Evans et al., 2016 [74] Di Pisa et al., 2019 [75]	Mowat-Wilson syndrome	Insomnia; Sleep–wake transition disorders.
Yaranagula et al., 2021 [76] Romigi et al., 2022 [77]	Hippocampal sclerosis	Worse subjective sleep quality; Sleep instability in polysomnographic studies.
Tezer et al., 2014 [78]	Hypothalamic hamartoma	Possible secondary hypersomnia.
Primiano et al., 2021 [79] Mosquera et al., 2014 [80]	Mitochondrial disorders	Sleep-disordered breathing.
Devine et al., 2022 [81]	LGI1/CASPR2 antibody encephalitis	Insomnia; Sleep-disordered breathing; REM behavioral disorder.
Ariño H et al., 2020 [82] Ribeiro et al., 2024 [83]	NMDAR antibody encephalitis	Insomnia at onset; Hypersomnia in the recovery phase; REM and NREM parasomnias.

Abbreviations: OSA—obstructive sleep apnea; REM—rapid eye movement sleep; NREM—non-rapid eye movement sleep.

**Table 4:** Effects of antiseizure medications on sleep and wakefulness and sleep parameters.

Authors	Antiseizure Medication	Impact on Sleep and Wakefulness	Sleep Strucure Alterations
Manconi et al., 2017 [127] Buscemi et al., 2007 [128] Kobayashi et al., 2025 [129]	Benzodiazepines	Excessive daytime sleepiness; NREM parasomnias.	↑ REM sleep latency; ↑ %1 NREM sleep; ↓ CAP rate.
Kwan et al., 2022 [130] Shen et al., 2017 [131] Wolf et al., 1984 [132]	Phenobarbital	Excessive daytime sleepiness.	↓ sleep latency; ↓ arousability; ↓ REM sleep; ↑ light sleep.
H. Zhang et al., 2014 [133] Javaheri et al., 2024 [134] Schmitt et al., 2009 [135]	Valproic acid	-	↓ of REM; ↑ N1 sleep; ↑ arousals; ↑ TST; Central sleep apnea patterns.
Wang et al., 2025 [136] Devinsky et al., 2019 [137] Patel et al., 2021 [138] Thiele et al., 2022 [139]	Cannabidiol	Excessive daytime sleepiness.	↑ SWS; ↑ REM sleep.
Nayak et al., 2016 [140]	Carbamazepine	Poor subjective sleep quality.	↑ CAP rate.
Thelengana et al., 2019 [141]	Oxcarbazepine	-	↑ TST.
Foldvary et al., 2001 [142] Placidi et al., 2000 [143]	Lamotrigine	-	↑ %REM sleep; ↓ SWS; ↑ 2 NREM sleep.
Lupo et al., 2023 [144]	Lacosamide	-	↑ SE; ↓ N1 sleep; ↓ N3 sleep.
Furey et al., 2014 [145] Kapustin et al., 2020 [146]	Gabapentin	Excessive daytime sleepiness.	↓ WASO; ↓ %N1 sleep; ↑ REM sleep.
Hindmarch et al., 2005 [147] Kapustin et al., 2020 [146]	Pregabalin	Excessive daytime sleepiness.	↑ SWS; ↓ sleep latency; ↑ SE.
Bazil et al., 2005 [148] Chaneva & Viteva, 2022 [149] Cicolin et al., 2006 [150] Zhou et al., 2012 [151]	Levetiracetam	Awakenings at night; Excessive daytime sleepiness.	↑ arousals; ↑ N2 sleep; ↓ N3 sleep; ↓ %REM sleep; ↑ TST, SE, SWS in some studies.
Rocamora et al., 2020 [152]	Perampanel	-	↑ TST; ↑ SE; ↑ SWS.

Abbreviations: REM—rapid eye movement sleep; NREM—non-rapid eye movement sleep; CAP—cyclic alternating pattern; TST—total sleep time; SE—sleep efficiency; SWS—slow-wave sleep; WASO—wake after sleep onset; N1, N2, N3—1, 2 and 3 stages of NREM sleep, respectively; ↓—decrease; ↑—increase.

## Data Availability

No new data were created or analyzed in this study.
